# Further Development of Near-Infrared Mediated Quantum Dots and Paclitaxel Co-loaded Nanostructured Lipid Carrier System for Cancer Theragnostic

**DOI:** 10.1177/1533033820914308

**Published:** 2020-04-27

**Authors:** Livesey D. Olerile

**Affiliations:** 1 The School of Pharmaceutical Sciences, Shandong University, Jinan, China

**Keywords:** co-loaded nanostructured lipid carrier, stability study, cellular uptake, theragnostic, translational system

## Abstract

Of colloidal systems, ceteris paribus, nanostructured lipid carriers are second to none in offering a single-unit platform for multifunctional benefits. Quantum dots are known to possess unique properties that make them ideal for imaging purpose and that they may be used for cancer detection. For several decades, paclitaxel has been the most effective drug against a wide range of solid tumours. Theragnostic nanomedicine provides a platform to monitor, evaluate, and individualize treatment in real time. Evaluation of cancer treatment outcome at an early stage therapy is key to increase survival prospects of a patient. Previously, a novel co-loaded nanostructured lipid carriers’ theragnostic system for parenteral administration was developed. The aim of this study was to further investigate the co-loaded nanostructured lipid carriers in order to provide interpretation necessary for preclinical elucidation of the formulation, in part. The co-loaded nanostructured lipid carriers were prepared by oil/water emulsification-solvent evaporation technique. In this study, stability and co-loaded nanostructured lipid carriers’ internalization by MCF 7 and HepG2 cells were investigated. The co-loaded nanostructured lipid carriers was stable at 4°C for 1 month. The formulation was successfully internalized by MCF-7 and HepG2 cells. Nevertheless, the co-loaded nanostructured lipid carrier was more apt for MCF-7 cells. This finding affirms the formulation to be the most appropriate for breast cancer treatment. In addition, if taken correctly by a patient for a month, the formulation would give true reflection of the contents’ amounts, the factor paramount to appropriate changes in treatment protocol. It can therefore safely be concluded that the co-loaded nanostructured lipid carrier formulation may be potentially an effective theragnostic translational system.

## Introduction

The development of nanocarriers for concomitant therapeutic and imaging applications has recently won considerable attention. This strategy potentially allows an approach that combines treatment and diagnosis in individual patient.^1-3^ Nanotheragnostic has evolved to become a promising strategy for personalized medicine.^4^ Theragnostic nanomedicine could provide unique features and new methodologies to monitor, evaluate, and individualize treatment in real time. Imaging agent may act as a tool to optimize individual patient dosage schedules and levels for the benefit of the patient and to evaluate treatment outcome at an early stage therapy by allowing individualized appropriate changes in treatment protocols, which are likely to increase the survival prospects for the patients.^5^

Developed at the turn of the millennium,^6^ nanostructured lipid carriers (NLCs) have improved characteristics in terms of drug loading and stability compared to solid lipid nanoparticles.^7^ Paclitaxel (PTX), a potent antineoplastic agent derived from the bark of pacific yew tree, *Taxus brevifolia*,^8,9^ is one of the most broadly active compounds being used against a wide spectrum of malignancies including cancers of breast, ovary, head and neck, and AIDS-related Kaposi’s sarcoma.^10-13^

Quantum dots (QDs) have highly sensitive fluorescent imaging properties with molar extinction coefficient as high as 0.5-5 × 10^6^ M^−1^·cm^−1^.^14^ This efficient photon absorption leads to nanomaterials that are 10 to 50 times brighter and several thousand times more photostable than conventional imaging dyes.^15^ Quantum dots offer a great promise as versatile probes integrating imaging and diagnosis. In light of bioimaging application, the qualities of wide absorbance range and narrow, highly symmetric emission spectra enable excitation of various sized QDs with a single wavelength resulting in distinct emission spectra with little overlapping.^16^ This reduces cross-talk between channels and allows improved multicolor detection, thereby enhancing detection efficiency and capability.

Imaging technique is chiefly limited by penetration depth due to the strong scattering properties of soft tissues.^17^ There is a strong scattering in the visible region of the spectrum (<700 nm). However, the near-infrared region ((NIR; 700-900 nm) often called “biological window” for optical imaging, is characterized by a low absorption and scattering in soft tissues.^18^ Nanoparticles with NIR excitation (650-900 nm) are highly preferable for *in vivo* imaging because of their penetration depth and minimized tissue autofluorescence compared with UV light.^19,20^ Thus, NIR QDs are suitable and more efficient for *in vivo* fluorescent imaging.

In previous study, a novel co-loaded NLC based on QDs and PTX^21^ to be used as a parenteral multifunctional delivery system was fabricated. This present study sought to further investigate the co-loaded NLC in order to provide interpretation necessary for preclinical elucidation of the formulation, in part. In doing so, short-term stability and internalization of co-loaded NLC by MCF-7 and HepG2 cells were investigated. It was hypothesized that the aforementioned investigations would augment the co-loaded NLC as an effective translation potential for cancer theragnostic.

## Materials and Methods

### Materials

Paclitaxel (purity >99%) was purchased from Shandong Chenxin Pharmaceutical Co, Ltd (Jinan, China). Quantum dots (CdTe/CdS/ZnS) were purchased from China Beijing Beida Jubang Science & Technology Co, Ltd (Beijing, China). Soya phosphatidylcholine (SPC) was provided by Shanghai Taiwei Pharmaceutical Co, Ltd (Shanghai, China). Glyceryl monostearate (GMS) was purchased from Tianjin Sitong Chemical Company (Tianjin, China), and oleic acid (OA) was purchased from Tianjin Damao Chemical Agents Company (Tianjin, China). Pluronic F68 (F68) was purchased from Sigma-Aldrich (St Louis, Missouri). Nonionic surfactant polysorbate 80 (Tween-80) was purchased from Sinopharm Chemical Reagent Co, Ltd (Shanghai, China). High-performance liquid chromatography (HPLC) grade acetonitrile was purchased from Cinc High Purity Solvents Co, Ltd (Shanghai, China); HPLC grade distilled water (Jinan, China) was used throughout the study. All chemicals and solvents were of analytical reagent or HPLC grade.

### Preparation of Co-loaded (PTX and QDs) NLC and Blank NLC

The desired amounts of GMS (37.5 mg), OA (14.03 µL), SPC (10 mg), PTX (3.5 mg), and 1.0 mL of 5 µM QDs were accurately weighed, measured, and quantitatively transferred into 2 mL eppendorf tube where they were dissolved in 1 mL of acetonitrile. The eppendorf tube was then submerged in a water bath at 80°C. The resulting organic phase was slowly (8 mL/h) injected by microsyringe pump (KD Scientific, Holliston, Massachusetts) into 10 mL of 0.5% wt/vol F68 aqueous solution, under mechanical agitation (RCT basic, Guangzhou, China) of 1000 rpm in a water bath at 80°C for 10 minutes to form a coarse emulsion. The warm primary coarse emulsion was further treated with a sonicator (>20 kHz) for 20 minutes to form a homogenous nanoemulsion. The resulting nanoemulsion was cooled down in an ice (0°C) bath to produce nanoparticle dispersion (PTX and QDs co-loaded NLC) and later stored at 4°C until use. The b-NLC was the one in which PTX was not added.^21^

### Physical Short-Term Stability Study of Co-loaded NLC

In determination of physical short-term stability of the co-loaded NLC formulation, the samples were stored in sealed amber colored glass vials at 4°C. After every week for 1 month of storage, the samples were evaluated for changes in particle size and encapsulation efficacy (EE).

For EE measurement, the desired amount of co-loaded NLC was dispersed in 2.9 mL of 0.5 wt% Tween 80-phosphate buffered saline (pH 7.4) and agitated (XW-80A vortex, Instruments factory of Shanghai Medical University, China) for 3 minutes to dissolve the free drug. The resulting dispersion was centrifuged at 2500 rpm (3-30 K Sigma, Henderson Biomedical Ltd, London, United Kingdom) for 10 minutes at 4°C. Upon centrifugation, the amount of the soluble free drug in the supernatant was harvested and measured by HPLC. The HPLC assay (Agilent 1100 series, USA) was performed on a reverse phase C18 analytical column (4.6 × 250 mm, pore size 5 μm, InertSustain, Tokyo, Japan). The mobile phase was a mixture of acetonitrile: water (65:35, vol/vol) delivered at a flow rate of 1.0 mL/min. Paclitaxel was detected at 227 nm with a variable wavelength detector. The calibration curve for quantification of PTX was linear (*R* ^2^ = 0.9988) over a range of standard concentrations between 1.0 and 50 μg/mL. The EE was calculated according to the following formula:

1 $$\text{EE}\left(\%\right)=\left[\left(W_{\text{total}}-W_{\text{free}}\right)/W_{\text{total}}\right]\times 100$$

For particle size evaluation, the mean particle size of the co-loaded NLC was established by photon correlation spectroscopy using Malvern Zetasizer (3000 HS, Malvern Instruments Ltd, Worcestershire, United Kingdom) at 25°C and 90° scattering angle. The samples were prepared in triplicates, measured, and averaged.

### Cell Maintenance and Cellular Uptake Study

The cancer cell lines of MCF-7 and HepG2 were obtained from Shandong Institute of Immunopharmacology and Immunotherapy of Shandong University (Jinan, China). The MCF-7 cells and HepG2 cells were cultured in Roswell park memorial institute (RPMI)-1640 medium and Dulbecco’s modified Eagle’s medium (DMEM), respectively. All the media were maintained with 10% (vol/vol) fetal bovine serum, penicillin (100 U·mL^−1^), and streptomycin (100 µg·mL^−1^). The cells were grown at 37°C in a humidified atmosphere containing 5% carbon dioxide.

For investigation of cellular internalization of the nanoparticle (co-loaded NLC), HepG2 and MCF-7 cell lines were utilized. In profile, HepG2 and MCF-7 cells were seeded (2 × 10^5^ cells per well) on coverslips in the 6-well plates each which included DMEM and RPMI-1640 medium, respectively. After 80% confluency, the medium was replaced with 1 mL of co-loaded NLC for 3 hours. After 3 hours of incubation, the cells were washed with phosphate-buffered saline (PBS; pH 7.4) and immediately fixed in 75% methanol and 25% glacial acetic acid for 15 minutes at 37°C. Thereafter, washed twice with PBS (pH 7.4), air dried, and stained with Hoechst 33342 (a DNA specific fluorescent dye) for 15 minutes. The stained coverslips (treated cells) were then observed under a laser scanning confocal microscope (Carl Zeiss LSM 700, Zeiss, Illinois).

### Statistical Analysis

All studies were independently repeated at least in triplicates, and data were presented as the mean ± standard deviation. Statistical analysis of differences among various treatments was carried out using the 2-tailed unpaired Student *t* test. Statistical significance was considered at *P* < 0.05.

## Results

### b-NLC and Co-loaded NLC Preparations

**Figure 1.**
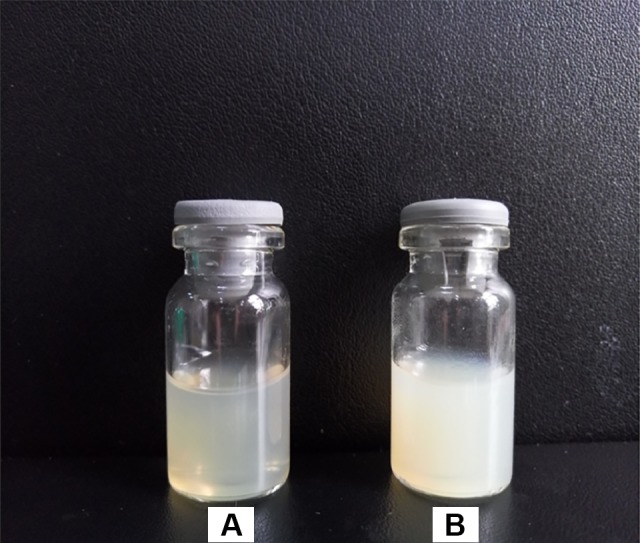
Representative photographs of (A) b-NLC and (B) co-loaded NLC. b-NLC indicates blank NLC; NLC, nanostructured lipid carrier.

Representative photograph of Figure 1A (b-NLC) showed clearer nanoparticle dispersion. Representative photograph of Figure 1B (co-loaded NLC) indicated almost the same nanoparticle dispersion as shown in Figure 1A except with white colouring from PTX. These results were not surprising because the method of preparation was the same for both preparations except that b-NLC was without PTX compared with the other.

### Stability Study of Co-loaded NLC

**Figure 2.**
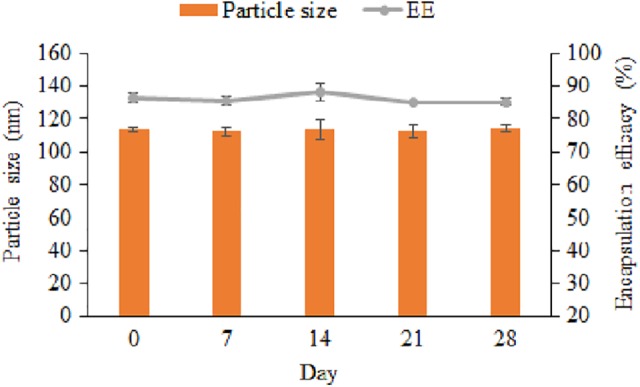
Stability study of co-loaded NLC as measured by particle size and encapsulation efficacy every week for 1 month (n = 3). The co-loaded NLC was stored at 4°C prior to analysis. NLC indicates nanostructured lipid carrier.

As depicted in Figure 2 (stability study of co-loaded NLC as measured by particle size and EE every week for 1 month), the particle sizes were found to be 113.60 ± 1.50 nm, 112.40 ± 3.02 nm, 113.90 ± 6.05 nm, 112.60 ± 4.17 nm, and 114.90 ± 2.26 nm, while EEs were found to be 86.59% ± 1.41%, 85.49% ± 1.25%, 87.81% ± 2.63%, 85.17% ± 0.56%, and 85.25% ± 1.07%, respectively. These results showed almost the same measurement for each of the measured parameters. By statistically comparing the 2 parameters, there were insignificant changes of the parameters.

### *In Vitro* Cellular Uptake of Co-loaded NLC

**Figure 3.**
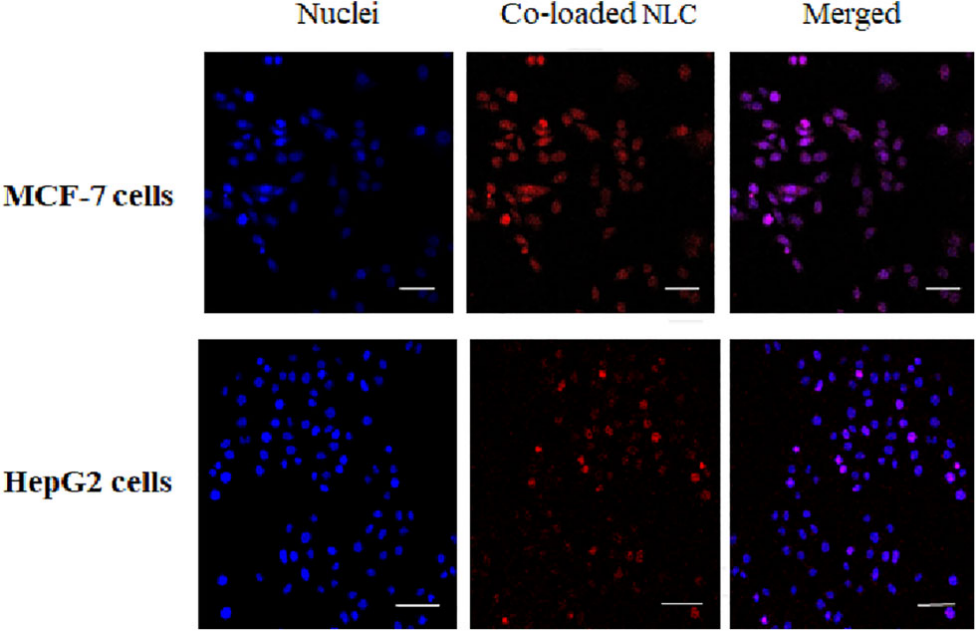
Internalization of co-loaded NLC by MCF-7 and HepG2 cells following exposure at 37°C for 3 hours. The cells nuclei were stained blue by Hoechst 33342 (excitation wavelength = 347 nm, emission wavelength = 483 nm) and overlaid with red fluorescent micrographs of co-loaded NLC (excitation wavelength = 675 nm, emission wavelength = 710 nm). All scale bars: 50 µm. Magnification: ×63. NLC indicates nanostructured lipid carrier.

Figure 3 shows internalization of co-loaded NLC by MCF-7 and HepG2 cells following exposure at 37°C for 3 hours. The nuclei of both MCF-7 cells and HepG2 cells were stained blue. Co-loaded NLC was stained red. The combination of blue and red colours makes purple (merged). With a high degree of confidence, it can be said that the co-loaded NLC reached the nucleus.

## Discussion

### b-NLC and Co-loaded NLC Preparations

By visual inspection, Figure 1, photographs of b-NLC and co-loaded NLC, indicates that the nanoparticles were very nearly transparent in appearance. This is consistent with hydrodynamic diameters ranging from 50 to 200 nm.^22,23^

### Stability Study of Co-loaded NLC

The purpose of stability testing is to provide proof on how the quality of active substance varies with time under the influence of a variety of environmental factors such as temperature and humidity. A short-term stability of co-loaded NLC dispersion was studied at 4°C every week for 1 month. Effects on particle size and EE were evaluated. The stability study of co-loaded NLC (Figure 2) revealed insignificant (*P* > 0.05) changes in the stability parameters. This invariability in both parameters (particle size and EE) suggests that the co-loaded NLC could remain stable for a month at 4°C. This indicates that in at least a period of month, the co-loaded NLC would present nearly the exact original amounts of contents. Consequently, a patient taking the right dose would have true reflection of the quantities of the dosage unit and hence appropriate treatment protocols be taken for the patient. Generally, water in formulation triggers instability overtime due to increased mobility of molecules. To avert this, the product is usually lyophilized using mannitol as a cryoprotectant.^24,25^ Although co-loaded NLC short-term stability was achieved for the period of 1 month, it is suggested that a lyophilized form of the formulation would be appropriate to cast-iron guarantee long-term stability.

### In *Vitro* Cellular Uptake of Co-loaded NLC

Cellular internalization of co-loaded NLC was investigated by the use of MCF-7 and HepG2 cell lines. It can be seen that the co-loaded NLC was successfully internalized by both MCF-7 and HepG2 cells (Figure 3). The results not only indicate that co-loaded NLC can enter the cell but also importantly that it reaches the nucleus (Hoechst 33342—a DNA specific fluorescent dye^26^). Considering 3-hour incubation period, it can be said that the internalization was fairly satisfactory since negatively charged nanoparticles prevent rapid cellular uptake due to repulsion emanating from negatively charged cellular membranes. In addition by visual inspection, internalization by MCF-7 cells looks clearer than that of HepG2 cells, probably due to the higher sensitivity of PTX to MCF-7 relative to HepG2 cells; the factor that may have led to PTX being approved by Food and Drug Administration as the first line of treatment for breast cancer.^27^ This finding affirms co-loaded NLC as the formulation appropriate for breast cancer treatment.

## Conclusion

The co-loaded NLC has demonstrated substantial short-term stability. The formulation was successfully internalized by MCF-7 and HepG2 cells. However, the co-loaded NLC was best suited for MCF-7 cells. This finding affirms the formulation to be the most appropriate for breast cancer treatment. It can therefore safely be concluded that the co-loaded NLC formulation may hold promise as an effective theragnostic translational system.

## Abbreviations

b-NLC: blank-nanostructured lipid carrier
DMEM: Dulbecco’s modified Eagle’s medium
EE: encapsulation efficacy
GMS: glyceryl monostearate
HPLC: High-performance liquid chromatography
NIR: near-infrared
NLC: nanostructured lipid carrier
OA: oleic acid
PBS: phosphate buffer solution
PTX: paclitaxel
QDs: quantum dots
RPMI: Roswell park memorial institute
SPC: soya phosphatidylcholine.
